# Mitochondrial Dysfunction in Huntington’s Disease; Interplay Between HSF1, p53 and PGC-1α Transcription Factors

**DOI:** 10.3389/fncel.2019.00103

**Published:** 2019-03-19

**Authors:** Taylor A. Intihar, Elisa A. Martinez, Rocio Gomez-Pastor

**Affiliations:** ^1^Department of Neuroscience, School of Medicine, University of Minnesota, Minneapolis, MN, United States; ^2^Department of Biochemistry and Molecular Biology, Dickinson College, Carlisle, PA, United States

**Keywords:** heat shock factor 1 (HSF1), mitochondrial dysfunction, Huntington (disease), p53, PGC-1α

## Abstract

Huntington’s disease (HD) is a neurodegenerative disease caused by an expanded CAG repeat in the huntingtin (*HTT*) gene, causing the protein to misfold and aggregate. HD progression is characterized by motor impairment and cognitive decline associated with the preferential loss of striatal medium spiny neurons (MSNs). The mechanisms that determine increased susceptibility of MSNs to mutant HTT (mHTT) are not fully understood, although there is abundant evidence demonstrating the importance of mHTT mediated mitochondrial dysfunction in MSNs death. Two main transcription factors, p53 and peroxisome proliferator co-activator PGC-1α, have been widely studied in HD for their roles in regulating mitochondrial function and apoptosis. The action of these two proteins seems to be interconnected. However, it is still open to discussion whether p53 and PGC-1α dependent responses directly influence each other or if they are connected *via* a third mechanism. Recently, the stress responsive transcription factor HSF1, known for its role in protein homeostasis, has been implicated in mitochondrial function and in the regulation of PGC-1α and p53 levels in different contexts. Based on previous reports and our own research, we discuss in this review the potential role of HSF1 in mediating mitochondrial dysfunction in HD and propose a unifying mechanism that integrates the responses mediated by p53 and PGC-1α in HD *via* HSF1.

## Introduction

Mitochondria are critical organelles that control energy production, lipid metabolism, and Ca^2+^ signaling and buffering. Decreased mitochondrial function has been implicated in multiple cellular processes, linking dysfunctional mitochondria to a wide range of human diseases, including metabolic, cardiovascular, and neurological disorders (Ballinger, 2005; Lin and Beal, 2006; Bhatti et al., 2017). The specific role of mitochondrial dysfunction in the context of Huntington’s disease (HD) has been the subject of numerous reviews in recent years (Quintanilla and Johnson, 2009; Reddy et al., 2009; Jin and Johnson, 2010; Oliveira, 2010; Costa and Scorrano, 2012; Dubinsky, 2017). Now, new findings have provided evidence for a novel role of Heat Shock transcription Factor 1 (HSF1) in directly regulating both mitochondrial function and HD pathology. Therefore, we discuss in this article the molecular mechanisms that contribute to mitochondrial dysfunction in HD and speculate on the possible role of HSF1 in mediating this defect.

HD is an inherited neurodegenerative disease caused by a CAG triplet (encoding glutamine) repeat expansion in the huntingtin (*HTT*) gene that causes HTT protein to misfold and aggregate (MacDonald, 1993; DiFiglia et al., 1997). HD is manifested by progressive behavioral and motor impairment accompanied by cognitive decline. In HD, striatal GABAergic medium spiny neurons (MSNs) are particularly vulnerable. Progressive dysregulation of MSNs is strongly correlated with motor symptoms onset and severity (Ferrante et al., 1991).

Altered mitochondrial morphology is a hallmark of HD and different abnormalities can be seen in different cell types. In peripheral tissues (lymphoblast, myoblast and fibroblasts) mitochondria present an enlarged morphology, while neurons are characterized by increased mitochondrial fragmentation (Panov et al., 2002; Squitieri et al., 2006, 2010; Kim et al., 2010; Jin et al., 2013). Altered mitochondrial structure correlates with mitochondrial dysfunction in all HD cells which is manifested by decreased electron transport chain activity, oxygen consumption, Ca^2+^ buffering and decreased ATP and NAD+ production (Oliveira, 2010). It has been proposed that mutant HTT (mHTT)-mediated mitochondrial abnormalities significantly affect MSNs due to the high-energy demand of this neuronal subtype (Ferrante et al., 1991; Pickrell et al., 2011). This is one hypothesis that explains the increased vulnerability of MSNs in HD (Ferrante et al., 1991; Mitchell and Griffiths, 2003). In support of this hypothesis, mitochondria isolated from the striatum of adult rats showed higher sensitivity to Ca^2+^ induced membrane permeabilization than mitochondria from the cerebral cortex, suggesting that striatal neurons are selectively vulnerable to metabolic stress (Brustovetsky et al., 2003). Other factors that contribute to this cell-selective neuropathology include; cell-type specific processing or localization of mHTT (Li et al., 2000; Menalled et al., 2002), abnormal interactions between mHTT and brain region specific protein partners and tissue specific differences in CAG instability (Kennedy et al., 2003; Goula et al., 2012). All these processes play important roles in promoting MSN degeneration, and although they could also contribute to increase mitochondrial stress, they are not the subject of this review.

HD patients and mouse models of HD exhibit well-described metabolic defects (Mochel and Haller, 2011; Mochel et al., 2012; Dubinsky, 2017). Metabolic analysis in presymptomatic patients using positron emission tomography (PET) and proton nuclear magnetic resonance (1H-NMR) showed that striatal glucose uptake and pyruvate utilization were reduced years before the onset of the motor symptoms, suggesting that mitochondrial alteration may be an early cause of disease progression (Antonini et al., 1996; Feigin et al., 2001). Other studies conducted in HD mouse models showed that MSN dendritic alterations appear even before mitochondrial respiratory defects can be observed, thus suggesting that energy deficits are a consequence of neuropathological changes (Guidetti et al., 2001). It is agreed that, either as a cause or as consequence, mitochondrial dysfunction is a key player in HD pathogenesis and progression. In recent years there has been a tremendous effort in developing therapeutic strategies towards improving mitochondrial function such as those aimed to stabilize mitochondria by boosting the production of ATP, decreasing membrane permeability and/or preventing oxidative damage (Reddy and Reddy, 2011; Corona and Duchen, 2016).

One additional function of mitochondria is to act as a reservoir for pro-apoptotic factors and therefore regulating cell death (Suzuki et al., 1999; Dumollard et al., 2009). Mitochondrial dysfunction, Ca^2+^ overload, and accumulation of reactive oxygen species (ROS) causes the mitochondrial permeability transition pore (mPTP) to open. mPTP opening triggers the intrinsic apoptotic pathway associated with the mitochondrial outer membrane permeabilization, cytochrome c release, and activation of caspase-3 (Choo et al., 2004; Quintanilla et al., 2017). The dysregulation of two main transcription factors p53 and PGC-1α has been extensively studied in HD for their roles in mediating mitochondrial dysfunction, apoptosis, and neurodegeneration (reviewed by Oliveira, 2010). We will briefly review these mechanisms of action and their crosstalk and discuss the potential role of HSF1 as a converging mechanism that integrates the responses mediated by p53 and PGC-1α.

## Role of p53 and PGC-1α in Mitochondrial Dysfunction

Transcriptional dysregulation and mitochondrial dysfunction are interconnected processes in HD governed by the crosstalk between p53 and the mitochondrial biogenesis factor PGC-1α (peroxisome proliferator-activated receptor γ co-activator 1α; Steffan et al., 2000; Jin and Johnson, 2010).

p53 is a transcription factor known for its role as a tumor suppressor through the regulation of several target genes with diverse biological functions including cell cycle arrest, DNA repair, metabolism, and apoptosis. p53 protein levels and activity are induced in the brain of HD patients and in cell and mouse models of HD^33^, explaining at least in part, the low tumor incidence observed in HD patients (Sørensen et al., 1999; Bae et al., 2005). mHTT strongly interacts with p53, and it has been proposed that such interaction impairs the recruitment of the E3 ligase Mdm2, thus increasing p53 stabilization (Steffan et al., 2000; Bae et al., 2005). Up-regulation of p53 leads to induced expression of different mitochondria associated proteins (e.g., Bax and Puma, linked to mitochondrial depolarization) and activation of apoptosis (Chipuk et al., 2004; La Spada and Morrison, 2005). The role of p53 in mediating mitochondrial dysfunction in HD was confirmed when primary neurons expressing mHTT were treated with the p53 inhibitor pifithrin-α and showed improved mitochondrial membrane potential (MMP) and increased cell viability (Bae et al., 2005). Recently, p53 was shown to also participate in mediating mitochondrial related necrosis and fragmentation in HD *via* direct interaction with mitochondrial fission protein Drp1 (dynamin related protein; Guo et al., 2013, 2014). However, the molecular mechanism by which p53 inhibition exerts neuroprotection is still poorly understood.

PGC-1α represents another major player in the link between mHTT, transcriptional dysregulation, and mitochondrial dysfunction (Johri et al., 2013). PGC-1α is a transcriptional coactivator that governs the expression of nuclear-encoded mitochondrial genes and regulates several metabolic processes, including mitochondrial biogenesis and oxidative phosphorylation (Wu et al., 1999; Puigserver and Spiegelman, 2003). Strikingly, PGC-1α null mice manifest HD-like features including, striatal neuronal loss, hypothermia and motor alterations (Weydt et al., 2006; Lucas et al., 2012). The expression of PGC-1α is significantly downregulated in MSNs compared to other striatal cells in HD patients and transgenic mouse models (Cui et al., 2006; Weydt et al., 2006). PGC-1α expression impairment in HD is due, at least in part, to the interference of mHTT with the CREB/TAF4 signaling pathway (Cui et al., 2006), which is considered the major regulator of PGC-1α expression (Herzig et al., 2001). However, chromatin immunoprecipitation analysis conducted in murine striatal-like cells derived from WT (ST*Hdh*^Q7^) and HD (ST*Hdh*^Q111^) mice did not show differences in CREB/TAF4 binding to the PGC-1α promoter between the two cell types (Cui et al., 2006) suggesting that additional mechanisms may be involved in PGC-1α expression impairment (discussed elsewhere in this review).

Down-regulation of PGC-1α in HD is accompanied by decreased expression of several PGC-1α–dependent targets and MSN markers (Weydt et al., 2006; Lucas et al., 2012). Studies aimed to induce the expression of PGC-1α in transgenic models of HD showed that PGC-1α promoted not only mitochondrial biogenesis but also provided neuroprotective effects by activating autophagy and increasing the turnover of mHTT aggregates (Tsunemi et al., 2012). These studies demonstrated the important role of PGC-1α in HD, and have motivated the generation of several pharmacological activators due to its therapeutic potential (reviewed by Johri et al., 2013).

However, recent transcriptomic analyses comparing different HD mouse models with either PGC-1α null mice or mice lacking PGC-1α in MSNs revealed many differences between their transcriptional profiles, particularly in mitochondrial-related genes (Lucas et al., 2012; McMeekin et al., 2018). Unexpectedly, HD knock-in mice showed up-regulation of several PGC-1α-dependent genes in an age-dependent manner. These data suggest that further studies in other mouse models will be necessary to clarify the exact role of PGC-1a in regulating mitochondrial gene dysregulation in HD.

Different reports have suggested that p53 and PGC-1α may operate together in controlling mitochondrial function, although the relationship between these two transcription factors differs depending on the physiological context. Studies in transgenic mice overexpressing the mitochondrial monoamine oxidase-A (MAO-A), an enzyme related to cardiomyopathies, showed that transgenic hearts exhibited p53 accumulation and downregulation of PGC-1α (Villeneuve et al., 2013), similar to what is observed in HD neurons. However, additional studies conducted in SH-SY5Y neuroblastoma cells upon glutathione shortage, showed that p53 binds to the PGC-1α promoter and positively regulates its expression (Aquilano et al., 2013), while in liver carcinoma cells Hep2G, p53, and PGC-1α proteins interact with each other and modulate their transactivation functions (Sen et al., 2011). These studies highlight the complexity in the regulatory mechanisms of these two transcription factors and open up the possibility to alternative regulatory pathways not yet described.

## HSF1 as a Physiological Regulator of Mitochondrial Activity

HSF1 is well known as the major transcriptional regulator of the heat shock response (Anckar and Sistonen, 2011). However, in the last decade a rising number of studies have proposed HSF1 to be a multifaceted factor involved in the regulation of many different cellular processes including but not limited to cell proliferation, inflammation, synapse formation, and energy metabolism (reviewed by Gomez-Pastor et al., 2017b). Here, we will discuss recent studies that have placed HSF1 in the spotlight for its role in mitochondrial function and neurodegeneration.

Benjamin and colleagues Yan et al. (2002) were the first to report a major role of HSF1 in regulating mitochondrial activity by studying the heart of Hsf1^−/−^ mice. Their studies showed that lack of HSF1 results in increased mPTP and increased ROS production. Additional studies in Hsf1^−/−^ oocytes confirmed the role of HSF1 in maintaining mitochondrial function and integrity by exhibiting mitochondrial ultrastructural abnormalities, functional defects, and activation of the apoptotic protein caspase-3 (Bierkamp et al., 2010). More recently, studies in Hsf1^−/−^ hepatocytes also revealed decreased ATP and NAD+ production and mitochondrial abnormalities attributed to altered Drp1 function (Qiao et al., 2017). However, whether these mitochondrial alterations were directly regulated by HSF1 or indirectly as a result of chaperone down-regulation is somewhat unclear.

Very elegantly, Nakai and colleagues Tan et al. (2015) showed that in primary mouse embryonic fibroblasts (MEFs) exposed to proteotoxic stress conditions, HSF1 recruits the mitochondrial SSBP1 factor (involved in replication of mitochondrial DNA) to the nucleus where they both co-operate to control the expression of several cytoplasmic/mitochondrial genes. Further studies in cancer cells also revealed that HSF1 directly regulates the expression of SMAC (mitochondria-derived activator of caspase) and other mitochondrial genes inhibiting mitochondrial apoptosis (Liang et al., 2017). These studies propose HSF1 as a novel mitochondrial responsive transcription factor (Lee et al., 2015).

Lack of HSF1 has also been associated with reduced neurogenesis, neuronal demyelination, and severe astrogliosis, leading to motor and cognitive deficits in aged mice (Santos and Saraiva, 2004; Homma et al., 2007; Uchida et al., 2011). Viability studies conducted in primary cortical astrocytes and neurons isolated from Hsf1^−/−^ mice exposed to different oxidative stress conditions revealed that both cell types were more sensitive than cells isolated from WT mice. Protein oxidation is also greater in Hsf1^−/−^ primary cultures (Homma et al., 2007). These results suggest that mitochondrial function could be impaired in different cell types in the brain of Hsf1^−/−^ mice, although no reports have addressed this issue yet. Whether the neuronal effects observed in HSF1 null mice are caused by mere chaperone depletion or directly related to HSF1-dependent regulation of mitochondrial gene transcription remains uncertain.

Recent studies conducted in adipose tissue revealed that HSF1 directly activates PGC-1α transcription by binding to a non-canonical Heat Shock Element (HSE) identified in its promoter sequence (Ma et al., 2015). This study highlighted the potential role of HSF1 in directly regulating mitochondrial function *via* regulation of PGC-1α. It is known that adipose tissue from HSF1 null-mice display mitochondrial gene expression deficits (Ma et al., 2015). However, lack of transcriptional studies in those cells impedes to determine whether lack of HSF1 specifically affects PGC-1α -dependent gene expression. In order to answer that question, further studies comparing the transcriptional profiles of HSF1 null mice and PGC-1 null mice will be necessary.

On the other hand, HSF1 and PGC-1α proteins interact and co-localize on several HSF1 target promoters co-operating in the regulation of different genes under hyperthermia (Xu et al., 2016). In fact, PGC-1α null cells showed down-regulation of several heat shock proteins, similar to those observed in HSF1-null mice (Trinklein et al., 2004; Xu et al., 2016). Intriguingly, PGC-1α also acts as a repressor of HSF1-mediated transcriptional program in hepatocytes and in cancer (Minsky and Roeder, 2015). Therefore, despite the evidence demonstrating the crosstalk between HSF1 and PGC-1α, the regulatory mechanisms that control PGC-1α and HSF1 interactions in different cell types or disease conditions is unclear.

## HSF1 Role in Mediating Mitochondrial Dysfunction in HD

HSF1 plays a fundamental role in HD pathogenesis (recently reviewed by Gomez-Pastor et al., 2017b). Studies where HSF1 null mice were crossbred with the R6/2 mice revealed that lack of HSF1 worsens neurodegeneration and disease progression (Hayashida et al., 2010) while HD transgenic mice overexpressing a constitutive active form of HSF1 significantly ameliorated HD symptoms (Fujimoto et al., 2005). The levels of HSF1 and its activity are strongly depleted in the striatum of patients with HD and in cell and mouse models of HD (Hay et al., 2004; Labbadia et al., 2011; Chafekar and Duennwald, 2012; Riva et al., 2012; Maheshwari et al., 2014; Gomez-Pastor et al., 2017a). HSF1 depletion is caused by inappropriate up-regulation of MSNs in two proteins, the Protein Kinase CK2α’ and E3 ligase Fbxw7, that phosphorylate and ubiquitylate HSF1, respectively, signaling the protein for proteasomal degradation (Gomez-Pastor et al., 2017a). It is believed that decreased levels and activity of HSF1 contribute to neuronal dysfunction and pathogenesis, suggesting HSF1 as a potential therapeutic target for HD intervention (Sittler et al., 2001; Neef et al., 2011). This hypothesis is supported by CK2α’ allele knock-out studies in the HD KIQ175 mouse model, which resulted in increased HSF1 levels and neuronal chaperone expression, rescued MSNs morphology and synapse formation, and ameliorated weight loss associated to HD (Gomez-Pastor et al., 2017a).

**Figure 1:**
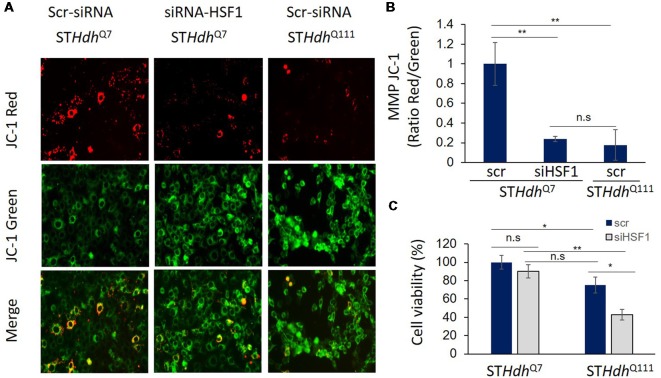
Heat Shock transcription Factor 1 (HSF1) regulates mitochondrial membrane potential (MMP) in striatal cells. **(A,B)** Murine immortalized striatal S*THdh*^Q7^ and ST*Hdh*^Q111^ cells were transfected with scramble (Scr.; Santa Cruz, sc-37007) or 5 μM siHSF1 (Santa Cruz, sc-35612). After 24 h, cells were incubated with 5 μM JC-1 dye (Invitrogen T3168) for 30 min at 37°C in PBS. MMP was determined by measuring fluorescence intensity at excitation 550 nm, emission 600 nm for red fluorescence (energized mitochondria) and at excitation 485 nm and emission 535 nm for green fluorescence (depolarized mitochondria). Representative fluorescence images are shown. Ratio red/green fluorescence was calculated for each condition and levels were relative to S*THdh*^Q7^ cells. A total of three independent experiments were performed. **(C)** Cell viability was quantified using CYQUANT XTT (ThermoFisher, X12223) after 24 h of transfection with scr. or siHSF1 following manufacturer’s instructions. Statistical analyses were performed using *T*-test, *p-value < 0.05, **p < 0.01, n.s. (no significant).

Due to previous studies linking HSF1 to mitochondrial function and PGC-1α expression (described above), it is reasonable to hypothesize that depletion of HSF1 could also contribute to the mitochondrial dysfunction and abnormalities reported in HD. Current research in our lab supports this hypothesis. We present here unpublished data evaluating the effects of silencing HSF1 in the MMP of murine striatal S*THdh*^Q7^ cells and how MMP alterations under these conditions mirror the deficits observed in scramble ST*Hdh*^Q111^ treated cells (Figure 1). The effect on MMP was determined using the JC-1 assay, a fluorescence dye that distinguishes between energized (JC-1 red) and depolarized (JC-1 green) mitochondria. We observed increased mitochondrial depolarization (determined by the ratio JC-1 red/JC-1 green signals) in ST*Hdh*^Q111^ compared to ST*Hdh*^Q7^ cells, as previously described (Ruan et al., 2004; Figure 1A). More importantly, silencing HSF1 in ST*Hdh*^Q7^ cells resulted in a strong mitochondrial depolarization compared to scramble, similar to the results obtained in untreated ST*Hdh*^Q111^ cells. Cell viability analyses using CyQUANT XTT assay (Thermo Fisher X12223) demonstrated that decreased MMP in ST*Hdh*^Q7^ cells treated with siHSF1 is not secondary to cell death since no significant changes were observed between scramble and siHSF1 conditions (Figure 1B). On the contrary, cell viability was reduced in ST*Hdh*^Q111^ compared to ST*Hdh*^Q7^ cells, as previously described using similar assays (Singer et al., 2017). This defect was exacerbated upon silencing HSF1. This data suggests that mitochondrial dysfunction contributes to exacerbating the HD phenotype although it is not sufficient to cause cell death.

**Figure 2:**
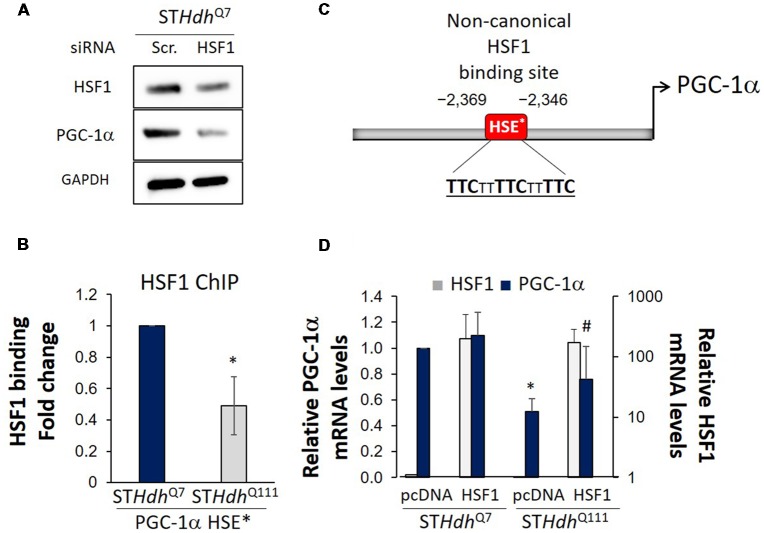
HSF1 binds to PGC-1α promoter and regulates its expression in Huntington’s disease (HD) cells. **(A)** Western blot analysis in S*THdh*^Q7^ cells treated with scramble (Scr.) or siRNA for 24 h. Cell lysates were prepared in lysis buffer (25 mM Tris pH 7.5, 150 mM NaCl, 1 mM EDTA, 1% Triton X-100, 0.1% SDS) and a total of 25 μg protein was loaded. **(B)** Diagram of PGC-1α promoter containing the non-canonical Heat Shock Element (HSE*; Ma et al., 2015). **(C)** HSF1 chromatin immunoprecipitation in S*THdh*^Q7^ and ST*Hdh*^Q111^ cells using 1 μg anti-HSF1 antibody (Bethyl Laboratories, A303-176A) and primers described by Ma et al. (2015; forward TTCATGGATGTGCTGGGTTA, reverse TTACAGATGGTTGCTTGCACT) for the PGC-1α promoter (Ma et al., 2015). Obtained values were normalized using % of input and fold enrichment over IgG (negative control) for each strain. Data was then expressed as fold change binding relative to S*THdh*^Q7^ cells. **(D)** qRT-PCR for PGC-1α expression (forward ATGTGTCGCCTTCTTGCTCT, reverse ATCTACTGCCTGGGGACCTT) performed 36 h after transfection with empty-pcDNA or HSF1-pcDNA overexpressing plasmid. At least three independent experiments were carried out for each analysis. Statistical analyses were performed using *T*-test, *p-value < 0.05. *p-value < 0.05 (compared to STHdhQ7-pcDNA), ^#^*p*-value < 0.05 (compared to STHdhQ7-HSF1).

Decreased MMP in ST*Hdh*^Q7^ cells treated with siHSF1 was accompanied by a decrease in the levels of PGC-1α (Figure 2A). We then conducted HSF1 chromatin immunoprecipitation analysis on the promoter of PGC-1α. We demonstrated that HSF1 binds to the non-canonical HSE present in the promoter of PGC-1α in both ST*Hdh*^Q7^ and ST*Hdh*^Q111^ cells (Figures 2B,C). However, HSF1 binding was reduced in ST*Hdh*^Q111^ cells (Figure 2C) correlating with the previously reported depletion of HSF1 and the reduced expression of PGC-1α in those same cells (Cui et al., 2006; Chafekar and Duennwald, 2012; Gomez-Pastor et al., 2017a). In line with HSF1 playing a role in the regulation of PGC-1α, overexpression of HSF1 in ST*Hdh*^Q111^ cells rescued the expression of PGC-1α (Figure 2D). These results suggest that depletion of HSF1 protein levels in HD (Gomez-Pastor et al., 2017a) could be responsible, at least in part, for the expression impairment of PGC-1α. In support of this hypothesis, we have previously observed that increasing HSF1 levels in the striatum of HD mice elevated the expression of PGC-1α and its downstream targets such as the cytochrome c and the mitochondrial transcription factor TFAM (Gomez-Pastor et al., 2017a). All this data provides evidence for the role of HSF1 degradation in contributing to mitochondrial dysregulation in HD. However, further experiments *in vivo* will be necessary to establish the direct connection between HSF1 depletion, mitochondrial impairment and PGC-1α down-regulation in HD.

## Crosstalk Between HSF1 and p53-PGC1α Axis

**Figure 3:**
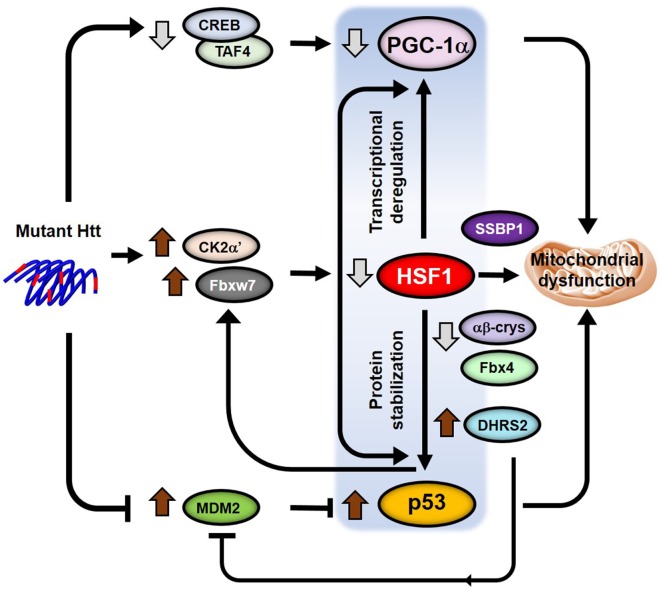
Model for p53-HSF1-PGC1α integrated responses in HD. Crosstalk between the transcription factors p53, HSF1 and PGC-1α regulate transcription, protein homeostasis, mitochondrial function and apoptosis. Different pathways (CREB/TAF4, CK2α’/Fbxw7 and Mdm2) are altered in the presence of mutant HTT (mHTT) that independently lead to the deregulation of the levels and functions of all three transcription factors. However, HSF1 becomes a key player in the subsequent regulation of the levels of both p53 and PGC-1α by directly regulating transcription of PGC-1α and controlling p53 protein stability in HD. The potential role of p53 in the regulation of the HSF1 degradation pathway in HD would add a positive feedback into the p53-HSF1-PGC-1α axis triggering mitochondrial dysfunction and neuronal death.

Different reports have revealed HSF1 crosstalk with the p53 pathway by directly regulating p53 stabilization and nuclear translocation (Li et al., 2008; Jin et al., 2009; Logan et al., 2009; Oda et al., 2018; Figure 3). In human diploid fibroblasts, acute depletion of HSF1 induces cellular senescence independent of chaperone-mediated protein homeostasis but dependent on activation of the p53-p21 pathway. This is partly because of the increased expression of dehydrogenase/reductase 2 (DHRS2), a putative MDM2 inhibitor. MDM2 regulates p53 degradation and its inhibition contributes to increased p53 levels and activation of apoptosis (Oda et al., 2018). A different study also reported increased levels of p53 in Hsf1^−/−^ MEFs (Jin et al., 2009). However, the authors proposed an alternative HSF1 dependent mechanism for the up-regulation of p53 levels. They showed that αβ-crystallin, an HSF1-gene target, is necessary to recruit the E3 ligase Fbx4 that ubiquitylates p53 and controls p53 degradation. In the absence of HSF1, reduced levels of αβ-crystallin results in the stabilization of p53.

On the other hand, p53 has been shown to directly regulate the expression of the human E3 ligase Fbxw7, by binding to regulatory elements contained within the Fbxw7 coding sequence (Kimura et al., 2003; Mao et al., 2004). Since Fbxw7 is involved in HSF1 ubiquitylation (Kourtis et al., 2015; Gomez-Pastor et al., 2017a) it is possible that increased p53 levels in HD participates in the degradation of HSF1 by up-regulating Fbxw7 during disease progression. If this hypothesis is correct, this would establish a vicious cycle where depletion of HSF1 contributes to the stabilization of p53 levels, which in turn potentiates HSF1 degradation (Figure 3).

As we previously discussed, p53 and PGC-1α pathways are also interconnected processes where the levels of one factor influences the levels and activity of the other (Sen et al., 2011; Aquilano et al., 2013; Villeneuve et al., 2013). Considering all the evidence that connects HSF1, p53, and PGC-1α, we speculate on the existence of a p53-HSF1-PGC-1α axis that integrates transcriptional dysregulation and mitochondrial dysfunction into one single pathway (Figure 3). However, it will be necessary to conduct further research to put together all the pieces of the puzzle and connect these three transcription factors in the context of HD.

## Future Directions

Numerous studies now demonstrated the role of HSF1 in regulating mitochondrial dysfunction in different contexts including HD. However, many questions still remain unresolved. First, it would be necessary to uncover whether elevation of p53 is responsible for the degradation of HSF1 in HD and whether the neuroprotection exerted by p53 inhibition is indeed mediated *via* HSF1. On the other hand, the direct role of HSF1 in regulating the expression of PGC-1α in HD and the consequences of such regulation on mitochondrial dysfunction in neurons needs to be further validated in other systems. These studies will be critical to fully understand the causes and consequences of HSF1 degradation in HD and will help to develop more efficient therapeutic strategies for HD intervention.

## Data Availability

All datasets generated for this study are included in the manuscript.

## Author Contributions

TI conducted experiments and contributed with writing, preparing literature and reviewing the manuscript. EM conducted experiments shown in Figure 1 and contributed with reviewing the manuscript. RG-P contributed to the writing of the manuscript, preparing literature and designed the experiments shown in the manuscript.

## Conflict of Interest Statement

The authors declare that the research was conducted in the absence of any commercial or financial relationships that could be construed as a potential conflict of interest.
